# Preliminary Study on the Genetic Structure and Functional Candidate Genes of Grassland-Thoroughbreds Based on Whole-Genome Resequencing

**DOI:** 10.3390/ani15101462

**Published:** 2025-05-19

**Authors:** Wenqi Ding, Wendian Gong, Tugeqin Bou, Lin Shi, Yanan Lin, Xiaoyuan Shi, Zheng Li, Huize Wu, Manglai Dugarjaviin, Dongyi Bai, Yiping Zhao

**Affiliations:** 1 Key Laboratory of Equus Germplasm Innovation (Co-Construction by Ministry and Province), Ministry of Agriculture and Rural Affairs, Hohhot 010018, China; dingwenqi0331@gmail.com (W.D.); gongwendian1996@outlook.com (W.G.); tvgqin@gmail.com (T.B.); 19832607527@163.com (L.S.); linyanan@emails.imau.edu.cn (Y.L.); xiaoyuans2021@163.com (X.S.); lzheng0511@sina.com (Z.L.); whz020419@163.com (H.W.); dmanglai@163.com (M.D.); baidongyi1983@163.com (D.B.); 2 Inner Mongolia Key Laboratory of Equine Science Research and Technology Innovation, Inner Mongolia Agricultural University, Hohhot 010018, China; 3 Equus Research Center, College of Animal Science, Inner Mongolia Agricultural University, Hohhot 010018, China

**Keywords:** grassland-thoroughbreds, selection signal, whole-genome resequencing

## Abstract

Speed and endurance are the primary goals in racehorse breeding. The Grassland-Thoroughbred is a newly developed breed in northern China that combines speed, endurance, and environmental adaptability. However, current research on the genetic background of this breed and the genes associated with athletic performance remains limited. We conducted whole-genome resequencing on Mongolian (MG), Thoroughbred (TB), Xilingol (XL), and Grassland-Thoroughbred (CY) horses, generating 3813.74 Gb of clean data after quality control. The number of transitions was significantly higher than that of transversions. The SNPs were mainly located in intergenic regions, followed by intronic regions. Principal component analysis, population structure analysis, and phylogenetic tree results indicated that the CYs had a distinct genetic background from MGs, TBs, and XLs, but based on PCA and phylogenetic clustering, they showed greater genetic similarity to Thoroughbreds. Using fixation index (Fst) and nucleotide diversity ratio (π ratio) analyses between CYs and the other three horse populations, 70, 76, and 80 candidate genes were identified from the intersection of the two methods, respectively. A total of 179 candidate genes were obtained from the union of the three groups. Candidate genes associated with athletic performance (*ATF2*, *NDUFS7*, *PRKG1*, *IGFN1*, *MTOR*, *TTN*) and growth and development (*MTOR*, *IGFN1*, *COL21A1*, *NEDD4*, *PIEZO1*) were screened. These genes are related to athletic ability and developmental processes in the CY population. Our study reveals genomic information associated with important traits in Grassland-Thoroughbreds and identifies valuable candidate genes, laying a foundation for future breeding and trait association studies.

## Simple Summary

This study conducted whole-genome resequencing of Mongolian horses, Thoroughbreds, Xilingol horses, and Grassland-Thoroughbreds, generating 3813.74 Gb of high-quality data and identifying 22,630,118 SNPs, which were mainly distributed in intergenic regions. Analysis revealed significant genetic differences between Grassland-Thoroughbreds and the other horse breeds, with a closer genetic relationship to Thoroughbreds. Based on Fst and π ratio analyses, key candidate genes associated with athletic performance (*ATF2*, *NDUFS7*, *PRKG1*, *IGFN1*, *MTOR*, *TTN*) and growth and development (*MTOR*, *IGFN1*, *COL21A1*, *NEDD4*, *PIEZO1*) were identified. This research provides new insights into the genetic basis of important traits in Grassland-Thoroughbreds and lays a foundation for future breeding programs and trait association studies.

## 1. Introduction

Since ancient times, horses have played an important role in society. With the development of society, their uses have also been constantly changing. The horse industry in different countries and regions exhibits diverse and distinctive models, such as leisure riding, horse racing, and equestrian sports [1]. Among them, horse racing, as a leading sector, not only attracts large audiences from around the world but also drives the development of related industries. Horse racing and breeding are major industries in at least 71 countries, involving approximately 500,000 horses worldwide. The total prize money for horse racing exceeds EUR 3.3 billion [2], and prestigious races such as the Melbourne Cup, the Kentucky Derby, and the Japan Cup are internationally recognized events [3,4,5]. Horse racing is not only an ancient and spectacular sport but also a significant driving force for regional economic and cultural development. With the rapid growth of the global horse racing industry, breeding has become increasingly important within the sector. Developing native horse racing breeds in China can not only enhance the competitiveness of domestic horse racing but also contribute to regional economic and cultural development.

The Mongolian horse is one of the oldest and most representative grassland horse breeds in the world, renowned for its exceptional endurance, tolerance to rough forage, cold resistance, and strong disease resistance [6]. As an outstanding local breed in the Inner Mongolia region, the Mongolian horse is often used as a parent in crossbreeding programs. Among these, the Xilingol horse is a new breed developed using the Mongolian horse as the maternal line, through 35 years of systematic selective breeding [7]. The Thoroughbred is globally recognized as one of the top horse racing breeds due to its exceptional competitive ability in speed races. It holds the world record for various distances ranging from 800 to 5000 m, setting and maintaining the fastest speeds in these short-distance events.

In order to further improve athletic performance, conformation, and speed, Thoroughbreds were introduced in 1995 for crossbreeding with Xilingol horses. They are also commonly used as high-quality breeding material in crossbreeding programs [8,9]. After nearly 30 years of continuous selective breeding and performance screening, the breeding program successfully produced a group of offspring with exceptional athletic potential, temporarily named the Grassland-Thoroughbreds. Due to the unique sunflower brand mark on their hindquarters, these horses are also known as “Sunflower Horses”. As breeding efforts progressed, traditional phenotypic selection became insufficient to fully reveal the genetic basis behind the horses’ complex traits. To advance the breeding of the Grassland-Thoroughbred, whole-genome resequencing of the Grassland-Thoroughbred was conducted to explore its genetic background and athletic potential.

Selective markers are genomic regions that show signatures of selection, either natural or artificial, during species evolution. They serve as effective tools for studying the adaptability of livestock to different natural environments and for exploring the genetic mechanisms underlying phenotypic differences [10,11]. SNPs (Single Nucleotide Polymorphisms) are the most common types of selective markers [12]. They represent DNA sequence polymorphisms caused by variations in a single nucleotide at the genomic level. SNPs are one of the most abundant, widely distributed, and commonly inherited variations, accounting for over 90% of known polymorphisms [13,14]. The application of SNP markers provides important tools for identifying genetic variations associated with key traits such as movement, immunity, and development. With the advancement of sequencing technologies and improvements in bioinformatics methods, the detection of selective markers has been widely used to uncover genomic signatures left by domestication and artificial selection, as well as to identify genes associated with important traits [15].

With the continuous advancement and development of genome sequencing technologies, molecular marker-assisted breeding has become an important method for developing new breeds. This study applies whole-genome resequencing and comparative genomics to identify selection signals and candidate genes associated with athletic potential in the Grassland-Thoroughbred. This is the first whole-genome analysis of the Grassland-Thoroughbred, laying the foundation for a deeper understanding of the genetic structure of this breed and providing valuable data support for future molecular breeding.

## 2. Materials and Methods

### 2.1. Sample Collection

In total, 103 horses were included in this study, comprising 82 newly collected samples and 21 public dataset samples from NCBI SRA (Supplementary Table S1). The Grassland-Thoroughbreds used in this study were sourced from the Inner Mongolia Grassland-Thoroughbred Breeding Co., Ltd. (Inner Mongolia, China), which has a detailed breeding plan (Supplementary Figure S1). Based on pedigree records, individuals with close kinship were excluded, and blood samples were collected from 35 Grassland-Thoroughbreds (CYs). Additionally, 36 Xilingol horses (XLs) were sampled from the Baiyinxile Ranch in Xilingol League, and 11 Mongolian horses (MGs) were collected from the Xilingol Grassland in Inner Mongolia. Blood samples were collected by professional technicians from the jugular vein of each horse, placed into EDTA anticoagulant tubes, aliquoted, and rapidly frozen in liquid nitrogen. The samples were then stored at −80 °C to maintain quality for subsequent experiments. Furthermore, sequencing data from 8 MGs and 13 Thoroughbreds (TBs) were downloaded from the Sequence Read Archive (SRA) database of the National Center for Biotechnology Information (NCBI) to further investigate the population structure of the Grassland-Thoroughbred.

### 2.2. Library Construction

Genomic DNA was first extracted from all blood samples using the CTAB method and subsequently used for DNA library preparation. The extracted DNA had concentrations ranging from 73.06 to 238.52 ng/µL, as measured using an Agilent 5400 (Supplementary Table S2). A total of 1 µg of DNA from each sample was used as input for library preparation. Sequencing libraries were constructed using the NEBNext^®^ Ultra™ DNA Library Prep Kit for Illumina (NEB, Ipswich, MA, USA; Catalog No. E7370L), according to the manufacturer’s instructions. Genomic DNA was sheared to an average fragment size of approximately 350 bp by ultrasonic fragmentation. Subsequently, DNA fragments underwent end repair, A-tailing, and ligation with Illumina sequencing adapters, followed by PCR amplification to enrich the DNA fragments in the library. PCR products were purified using the AMPure XP system (Beckman Coulter, Brea, CA, USA), and library quality was assessed using the Agilent 5400 system (Agilent, Santa Clara, CA, USA). Libraries that met the requirements for effective concentration and data quality were submitted to Novogene Bioinformatics Technology Co., Ltd. (Beijing, China) for high-throughput sequencing on the Illumina HiSeq 4000 (Illumina, San Diego, CA, USA) platform using the PE150 strategy, generating 150 bp paired-end reads with an average sequencing depth of 10× per sample.

### 2.3. Quality Control and Data Filtering

We used fastp (v0.23.1) to perform quality control on the raw sequencing data and the data downloaded from NCBI to ensure the reliability of subsequent analyses. The quality control criteria included the following: (1) reads with ≥5% unidentified bases (N) were removed; (2) removal of reads in which more than 20% of bases had a Phred quality score below 15; (3) removal of reads containing adapter sequences, allowing ≤3% mismatches; and (4) removal of reads shorter than 15 bases [16]. The processed data were then evaluated using FastQC (v0.11.5) to assess quality and confirm their suitability for downstream analyses [17].

### 2.4. Variant Detection and Annotation

The clean data were aligned to the horse reference genome (https://www.ncbi.nlm.nih.gov/datasets/genome/GCF_002863925.1/, accessed on 27 November 2020) [18] using BWA (version 0.7.15). The resulting alignment files were then converted and sorted using Samtools (version 1.3). Picard tools (github.com/broadinstitute/picard, accessed on 25 August 2021) were used with the MarkDuplicates command, setting REMOVE_DUPLICATES = true to exclude potential PCR duplicates. On the recalibrated BAM files, SNP calling was performed using GATK HaplotypeCaller (version 4.0.3.0), followed by data merging with CombineGVCFs. The SelectVariants module was then used to extract the raw SNPs and raw Indels. The SNP file was filtered using the VariantFiltration parameter with the following criteria: QUAL < 30.0, QualByDepth (QD) < 1.5, RMS Mapping Quality (MQ) ≥ 4, and Depth of Coverage (DP) < 5. After applying these filters, a VCF file containing high-quality SNPs was generated. Further filtering was performed using VCFtools: SNPs missing in more than 80% of the samples were excluded (--max-missing 0.8), variants with a minor allele frequency (MAF) greater than 0.05 were retained (--maf 0.05), only biallelic variants were kept (--min-alleles 2--max-alleles 2), and sites with a quality score (QUAL) ≥ 30 were retained. SNPs not mapped to autosomes or located on sex chromosomes were excluded to ensure data accuracy and consistency [19].

### 2.5. Population Structure Analysis

Prior to analysis, all SNPs were trimmed with PLINK (version 1.9) software’s indep-pairwise [20], with parameters set to a non-overlapping window of 50 SNPs, a step size of 5 SNPs, and a threshold of 0.2 for r^2^ to obtain independent SNP markers. Principal component analysis (PCA) was performed on the obtained SNP dataset using PLINK. The first two principal components were plotted using the ggplot2 package (version 3.3.5) in R (version 3.6.1) to visualize the clustering patterns among different horse populations [21]. To evaluate the genetic relationships among individuals, a distance matrix was calculated using VCF2Dis (version 1.50). A neighbor-joining phylogenetic tree was then constructed using the neighbor program from the PHYLIP package (Version 3.697). The resulting tree was visualized and refined using the iTOL online tool (https://itol.embl.de/, accessed on 10 December 2024), providing a more intuitive representation of the genetic relationships and population structure among different horse breeds. In addition, to further assess the genetic composition and potential population stratification, the population structure was inferred using ADMIXTURE (version 1.3.0). A range of K values were tested, and the most likely K was determined based on the CV error. The results were visualized using TBtools (version 2.154). Linkage disequilibrium (LD) decay was estimated using PopLDdecay (version 3.42) based on the correlation coefficient (r^2^) of allele frequencies. Parameters were set as -MaxDist 1000 and -MAF 0.05. The -SubPop option was used to specify the population information file. Finally, the LD decay curves were plotted using the built-in Perl script Plot_MultiPop.pl provided with PopLDdecay.

### 2.6. Detection of Selection Signatures

Fst is an important metric in population genetics used to measure the degree of genetic differentiation among subpopulations, reflecting the differences in allele frequencies between groups. Fst generally ranges from 0 to 1, with higher values indicating greater differentiation and higher allele frequency divergence among populations. The population differentiation index was calculated using vcftools (version 0.1.15) with a sliding window approach [22]. This sliding window strategy enhances the sensitivity to target signals while reducing the probability of false positives. The sliding window size was set to 100 kb with a step size of 10 kb, using the parameters --fst-window-size 100,000 --fst-window-step 10,000. Genomic regions within the top 1% of Fst values were identified as candidate regions.

Nucleotide diversity (π) is an important indicator of the level of genetic variation within a population. It is calculated by measuring the base differences between the pairwise comparisons of individuals in a sample and determining the average nucleotide difference at the same locus across individuals. Essentially, the π value reflects the heterozygosity at each base position over a unit length of the sequence. The larger the π value, the higher the nucleotide diversity. Nucleotide diversity was calculated using vcftools with a sliding window approach, where the sliding window size was set to 100 kb, and the step size was set to 10 kb. The parameters used were --window-pi-step 10,000 --keep group1/group2. After calculating nucleotide diversity using the sliding window strategy, a Python (version 3.9) script was employed to compute the genetic diversity ratio (π ratio) between the two populations. Genomic regions within the top 1% of π ratio values were selected as candidate regions.

### 2.7. Functional Enrichment Analysis

Functional enrichment analysis can provide an in-depth understanding of the regulatory mechanisms of genes associated with horse traits, offering valuable directions for future research. To elucidate the functions and regulatory mechanisms of candidate genes, this study conducted functional enrichment analysis. The online tool DAVID (https://davidbioinformatics.nih.gov/, accessed on 12 January 2025) was used for Gene Ontology (GO) and Kyoto Encyclopedia of Genes and Genomes (KEGG) enrichment analysis of the candidate genes. To ensure the biological relevance of the enrichment results, only annotations with a *p* < 0.05 were retained.

## 3. Results

### 3.1. Sequencing Data and Genome-Wide Genetic Variation

**Figure 1:**
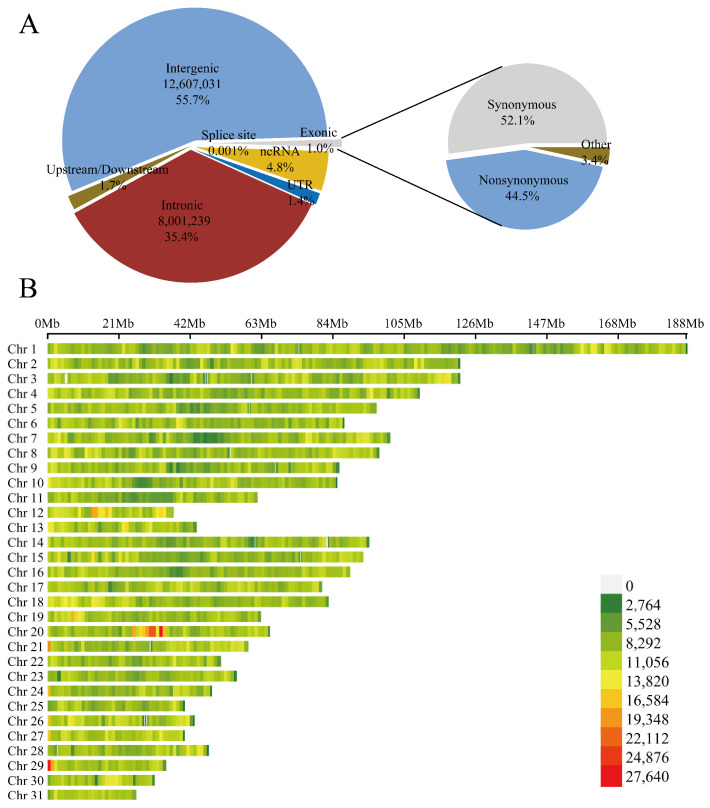
Genome-wide SNP annotation and chromosomal distribution map. (**A**) The annotation of genome-wide SNPs according to Annovar. (**B**) The SNP density across the whole genome was estimated in each 1 Mb genome block.

In this study, a total of 82 resequenced samples and 21 NGS data were used, generating 3813.74 Gb of clean data, with an average sequencing depth of 15.72× (Supplementary Table S3). A total of 22,630,118 SNPs were obtained in the analyzed population. Among these SNP sites, the number of transitions (A/G, C/T) was significantly higher than that of transversions (A/C, A/T, G/C, G/T). Annotation using ANNOVAR (https://annovar.openbioinformatics.org/en/latest/user-guide/download/, accessed on 12 June 2023) indicated that most variants were located in intergenic regions, followed by intronic regions, with a smaller proportion distributed in exonic regions, splice sites, and upstream or downstream gene regions (Figure 1A) (Supplementary Table S4). The number of SNPs on each chromosome was positively correlated with chromosome length, with chromosome 1 being the longest and containing the most SNPs, while chromosome 31 had the fewest (Figure 1B). In addition, Indels among the four populations were identified based on the resequencing data. The results showed that deletions outnumbered insertions across all populations, and the distribution pattern of Indels was consistent with that of SNPs (Supplementary Figure S2).

### 3.2. Population Structure Analysis

**Figure 2:**
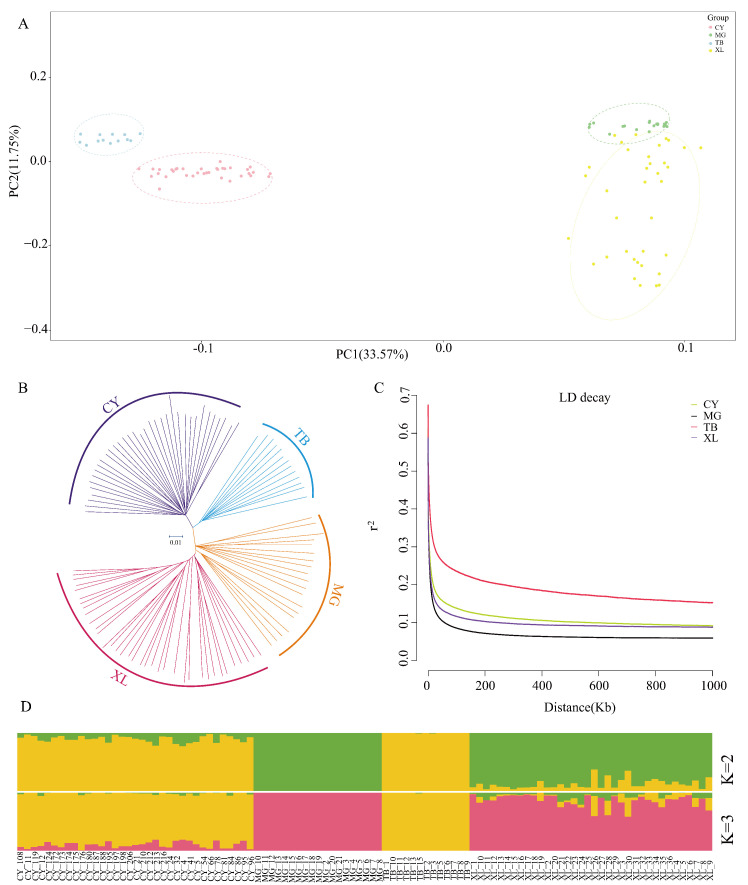
Population genetic structure analysis of four horse breeds. (**A**) Principal component analysis (PCA). (**B**) Phylogenetic tree (neighbor-joining tree, N-J tree). (**C**) Linkage disequilibrium (LD) decay levels. (**D**) Population structure analysis (different colors represent different ancestral components).

To understand the genetic relationships and differences among various horse breeds from a whole-genome perspective, we performed principal component analysis (PCA), phylogenetic tree construction, and population structure analysis on SNP datasets obtained from four horse populations. The PCA results showed that PC1 and PC2 explained 33.57% and 11.25% of the genetic variation, respectively. The results indicated that MGs and XLs exhibited a low degree of separation, while the XLs were more dispersed, suggesting a higher level of genetic variation within this group. The CYs were positioned between the XLs and the TBs, but closer to the TBs. Overall, the PCA revealed that each pair of populations was relatively close to one another, indicating similar genetic characteristics along the principal components (Figure 2A). To validate the results of the PCA, population genetic structure was inferred using the ADMIXTURE software (Version 1.3.0). When K = 2, the MGs and TBs each formed distinct ancestral genomic clusters, indicating substantial genetic differentiation between them. The XLs were more closely related to the MGs, with some individuals exhibiting similar genetic backgrounds. The CYs exhibited genetic admixture, with a high proportion of TB ancestry in their genomes, which was consistent with the PCA results. When K = 3, the XLs exhibit more complex variation characteristics, while with the continued hybridization of TBs, the CYs become closer to the TBs (Figure 2D). A phylogenetic tree was constructed using the neighbor-joining method. The results showed that samples from each population clustered together, demonstrating clear population separation (Figure 2B). This was consistent with the results of the principal component analysis and population structure analysis. The samples were grouped into four distinct clades. Linkage disequilibrium (LD) can reflect the intensity of the selection pressure experienced by a population. LD decay analysis showed that the TBs exhibited the slowest decay, which may indicate that they have been subjected to long-term and intensive artificial selection, resulting in higher levels of linkage disequilibrium. In contrast, the MGs showed the fastest LD decay, suggesting higher genetic diversity and long-term reproduction under natural conditions. The XLs and CYs showed intermediate decay rates, with the XLs decaying faster than the CYs. Although both populations have undergone artificial selection, the CYs may have been subjected to more stringent selective breeding (Figure 2C).

### 3.3. Selection Signal Analysis and Functional Enrichment

#### 3.3.1. Selection Signal Detection Between Grassland-Thoroughbred and Mongolian Horse

**Figure 3:**
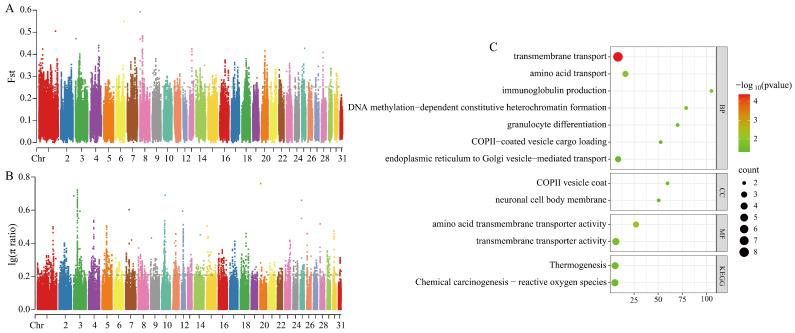
Selection signal analysis and functional enrichment. (**A**) Fst selection sweep analysis plot for CY vs. MG. (**B**) π ratio selection sweep analysis plot for CY vs. MG. (**C**) Candidate gene functional enrichment for CY vs. MG.

Selection signal analysis was conducted by integrating two methods: the Fst and π ratio. Genomic regions ranking within the top 1% of both metrics were defined as candidate selective regions. In the comparison between CYs and MGs, a total of 2259 candidate regions were identified using both approaches (thresholds: Fst = 0.251969; log_10_(π ratio) = 0.207659), corresponding to 600 and 816 genes (Figure 3A,B). A Venn diagram was used to identify overlapping genes between the two methods, yielding 70 candidate genes for further analysis.

GO analysis revealed significant enrichment in seven biological processes, two cellular components, and two molecular functions. KEGG analysis identified three significantly enriched signaling pathways (*p* < 0.05) (Figure 3C). The enriched GO terms included transmembrane transport (amino acid transport GO:0006865, transmembrane transport GO:0055085; transmembrane transporter activity GO:0022857) and immune-related functions (immunoglobulin production GO:0002377). KEGG pathways were significantly enriched in thermogenesis (ecb04714) and chemical carcinogenesis–reactive oxygen species (ecb05208).

#### 3.3.2. Selection Signal Detection Between Grassland-Thoroughbred and Thoroughbred Horse

**Figure 4:**
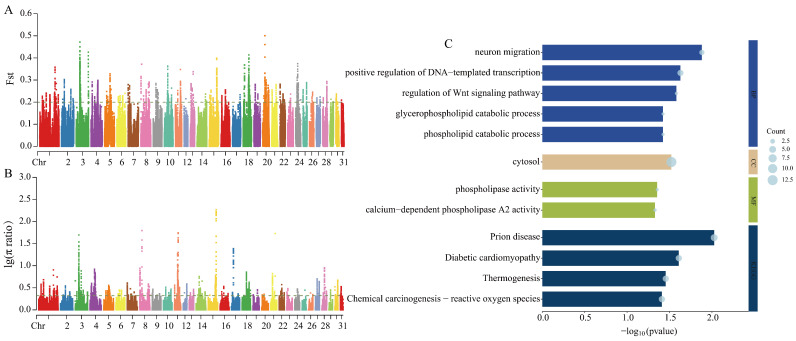
Selection signal analysis and functional enrichment. (**A**) Fst selection sweep analysis plot for CY vs. TB. (**B**) π ratio selection sweep analysis plot for CY vs. TB. (**C**) Candidate gene functional enrichment for CY vs. TB.

A selective sweep analysis between CYs and TBs identified 565 and 561 genes using Fst and π ratio scans, respectively (thresholds: Fst = 0.199512, log_10_(π ratio) = 0.322316). A total of 76 candidate genes were identified by both methods (Figure 4A,B). These candidate genes were significantly enriched in 9 GO terms and 4 KEGG pathways. GO functional enrichment was predominantly related to lipid metabolism (phospholipid catabolic process GO:0009395; glycerophospholipid catabolic process GO:0046475; phospholipase activity GO:0004620), neuronal migration (GO:0001764), and development (regulation of Wnt signaling pathway GO:0030111). KEGG analysis revealed significant enrichment in thermogenesis (ecb04714) and disease-associated signaling pathways (ecb05020; ecb05415; ecb05208) (Figure 4C).

#### 3.3.3. Selection Signal Detection Between Grassland-Thoroughbred and Xilingol Horse

**Figure 5:**
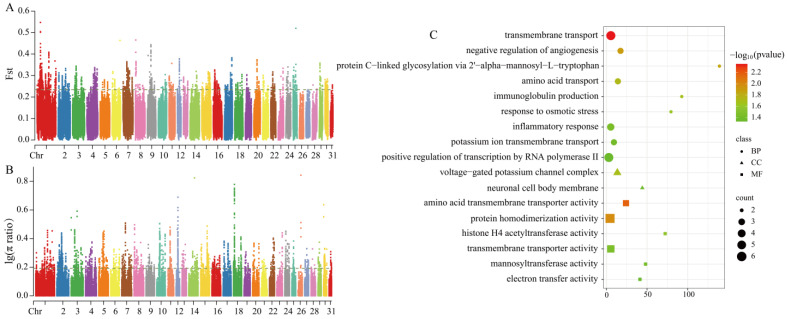
Selection signal analysis and functional enrichment. (**A**) Fst selection sweep analysis plot for CY vs. XL; (**B**) π ratio selection sweep analysis plot for CY vs. XL; (**C**) Candidate gene functional enrichment for CY vs. XL.

Using the same methods, a total of 83 candidate genes were identified between the CYs and XLs (thresholds: Fst = 0.234576, log_10_(π ratio) = 0.192149) (Figure 5A,B). GO functional enrichment analysis revealed 17 significantly enriched signaling pathways, primarily associated with transmembrane transport (transmembrane transport GO:0055085; amino acid transport GO:0006865; potassium ion transmembrane transport GO:0071805, amino acid transmembrane transporter activity GO:0015171; transmembrane transporter activity GO:0022857; voltage-gated potassium ion channel complex GO:0008076) and electron transport (GO:0009055) (Figure 5C) (Supplementary Table S6).

#### 3.3.4. Candidate Genes Under Selection in the Grassland-Thoroughbred

By comparing the CYs with MGs, TBs, and XLs, a total of 179 candidate genes under positive selection were identified through union extraction and screening (Supplementary Table S5). A further analysis of these genes led to the identification of candidate genes associated with athletic performance (*ATF2*, *NDUFS7*, *PRKG1*, *IGFN1*, *MTOR*, *TTN*) and those associated with growth and development (*MTOR*, *IGFN1*, *COL21A1*, *NEDD4*, *PIEZO1*).

## 4. Discussion

Horses occupy an important position among livestock. In the development of modern society, horses have integrated into human life in various ways and hold a significant role in the leisure industry. The main purpose of breeding is to selectively breed horses based on their performance characteristics (speed, endurance, strength, gait), appearance (size, color, structure), and temperament, resulting in the formation of 400–500 different horse breeds [23]. The Grassland-Thoroughbred was developed based on Mongolian horses, Xilingol horses, and Thoroughbreds, and its genetic background is closely related to these three groups. Among them, Mongolian horses exhibit the highest genetic diversity, which may be attributed to long-term natural selection and extensive genetic exchange. Thoroughbreds show the lowest level of genetic variation, likely due to reduced genetic diversity caused by high levels of inbreeding. As a cultivated breed, the Grassland-Thoroughbred displays a level of genetic variation between that of Xilingol horses and Thoroughbreds, possibly reflecting the characteristics of a hybrid breed that retains part of the genetic diversity of Mongolian and Xilingol horses (Supplementary Table S4). PCA revealed that the Grassland-Thoroughbred population is genetically closer to the Thoroughbred population, which may be due to continuous backcrossing with Thoroughbreds. ADMIXTURE and phylogenetic tree analyses further confirmed the results of the clustering analysis. The Xilin Gol horse, as a complex hybrid breed formed by the crossbreeding of various types of horses, may lead to its complex genetic traits, with some characteristics being inherited by the steppe purebred horses. In addition, a genomic analysis was used to explore the similarities and differences among these populations in terms of genetic diversity, athletic performance, and breeding potential. Fst can be used to measure the degree of genetic differentiation between populations, while the π ratio allows for the comparison of genetic diversity across populations. These methods have been widely applied in studies on the molecular genetic mechanisms underlying livestock domestication and the formation of important economic traits.

In the process of horse breeding, athletic performance is a highly complex and important trait that is closely associated with multiple factors. With the continuous advancement of genomic technologies, an increasing number of candidate genes related to athletic traits have been identified, making this a key research area for improving equine performance. Among them, *ATF2* was enriched in thermogenesis in both KEGG analyses. During acute exercise in mice, the phosphorylation levels of p38 MAPK and *ATF2* were significantly elevated [24]. p38 MAPK is an essential enzyme for myoblast differentiation and plays a role in glucose metabolism and energy expenditure. When exercise is induced, the p38 MAPK pathway is activated. As a downstream target of p38 MAPK, *ATF2* is involved in the p38 MAPK-mediated regulation of PGC-1α, which plays a crucial role in skeletal muscle adaptation [25]. Grassland-Thoroughbreds must run and train over long distances and in temperature-variable grassland environments, so efficient thermogenesis and energy regulation are vital for their adaptability. The enrichment of *ATF2* suggests that it may be an important regulatory factor for enhancing endurance and environmental adaptability in this breed. *NDUFS7* is a subunit of one of the mitochondrial respiratory chain complexes, known to enhance mitochondrial function and improve insulin sensitivity. Compared to young rats, the expression level of mitochondrial biogenesis-related *NDUFS7* is significantly dysregulated in aged rats. Under high-intensity exercise conditions, the expression level of *NDUFS7* is higher than that of the control group, indicating that *NDUFS7* plays a role in optimizing energy metabolism in skeletal muscle and the neuromuscular system [26,27]. Grassland-Thoroughbreds demonstrate the ability to sustain high-intensity output during training and competition, indicating that their muscle tissue possesses an efficient energy conversion mechanism. The high expression of *NDUFS7* may serve as a core foundation supporting this sustained athletic performance. *PRKG1* showed signs of positive selection in Grassland-Thoroughbreds across all comparison groups. Similarly, *PRKG1* (cGMP-dependent protein kinase 1) is widely expressed in the nervous system and is believed to play a critical role in neural plasticity and learning ability [28]. Studies have shown that *PRKG1* expression is significantly upregulated under high-intensity interval training conditions [29]. This gene is also considered a potential selection marker for memory and learning ability in Thoroughbreds [30] and may have potential applications in enhancing athletic performance and training adaptability in horses. The positive selection of *PRKG1* in the genome may enhance behavioral responsiveness to high-intensity training, leading to improved training outcomes and greater adaptability. Muscle strength is generally considered to be closely associated with overall health. *IGFN1* (Immunoglobulin-like and Fibronectin Type III Domain Containing 1) is more highly expressed in young individuals and gradually decreases with age. Additionally, *IGFN1* expression is upregulated in individuals who perform well on the “Timed Up and Go” test [31]. Studies have also found that the mRNA expression level of *IGFN1* in muscle increases after exercise training [32]. Endurance exercise can enhance insulin sensitivity and induce specific adaptations in skeletal muscle. The activity of *MTOR* is regulated by repeated contractions (exercise) and has been identified as a key mediator of exercise-induced skeletal muscle adaptation. During both aerobic endurance exercise and high-intensity resistance training, the activation level of *MTOR* rises sharply within a few hours post-exercise [33]. Long-term resistance training may lead to sustained increases in *MTOR* activity, helping to maintain skeletal muscle mass while inhibiting muscle degradation and atrophy [34]. Additionally, the T allele at rs10497520 in the *TTN* (titin) gene has been associated with shorter skeletal muscle fascicle length and has shown advantages among trained male marathon runners [35]. Grassland-Thoroughbreds exhibit pronounced muscle definition and contractile strength, suggesting an advantage in muscular structure. *TTN*, *MTOR*, and *IGFN1* all contribute to the optimization of skeletal muscle structure and strength. The functions of these genes are closely aligned with the actual performance of Grassland-Thoroughbreds in competition, potentially indicating a genetically favored foundation at the molecular level that supports adaptation to the demands of athletic performance.

Muscle development is often regarded as a key indicator for evaluating athletic potential and training effectiveness. Previous studies on the skeletal muscles of Grassland-Thoroughbred horses, Mongolian horses, and Xilingol horses have shown that Grassland-Thoroughbreds possess a significantly higher proportion of fast-twitch muscle fibers and a body type more similar to that of Thoroughbreds, leaning toward a racing phenotype, which may be closely related to their muscle development [36]. This study identified several key genes involved in the regulation of muscle development, suggesting a genetic basis for their muscle growth. The mammalian target of rapamycin (*MTOR*) is a serine/threonine kinase that regulates muscle cell growth and myogenesis [37]. *MTOR* can be activated by various stimuli, including insulin, growth factors, and amino acids. In skeletal muscle, MTOR promotes the proliferation of satellite cells through the mediation of *WNT7A* and contributes to muscle fiber growth [38]. *IGFN1* is an essential gene involved in myoblast fusion and differentiation [39]. Functional association analysis has revealed that *IGFN1* is primarily involved in processes such as protein translation, skeletal muscle contraction, muscle development, cytoskeletal organization in skeletal muscle cells, and intracellular calcium homeostasis [40]. Studies have shown that *IGFN1* may regulate myoblast fusion by influencing actin remodeling through its interaction with *COBL* [41]. *COL24A1* is a fibrillar collagen primarily expressed in the skeleton during mouse embryogenesis, as well as in the trabecular bone and periosteum of newborn mice. Its expression level gradually increases during the progression of osteoblast differentiation. *COL24A1* may regulate osteoblast differentiation by interacting with integrin β3 and modulating the TGF-β/Smads signaling pathway [42]. *NEDD4*, a ubiquitin ligase, is expressed in muscle satellite cells and acts as a novel regulatory factor of *PAX7*. It interacts with *PAX7* during early muscle differentiation, where the accumulation of *NEDD4* leads to a decrease in *PAX7* levels and promotes premature muscle cell differentiation [43]. In muscle stem cells, the expression level of *PIEZO1* is significantly reduced, resulting in impaired myogenic potential. This process may involve the suppression of myogenic capacity through the ERK and MAPK signaling pathways, while the *PIEZO1* activator *YODA1* may help alleviate muscle degeneration [44]. As a sport-oriented breed, the robust muscle development and superior athletic performance of the Grassland-Thoroughbred may be closely associated with the coordinated regulation of genes, such as *MTOR*, *IGFN1*, *COL24A1*, *NEDD4*, and *PIEZO1*, which are involved in muscle cell proliferation, differentiation, fusion, and skeletal development.

The genetic variation analysis based on whole-genome resequencing in this study is still in the preliminary stage. Although high-depth sequencing and strict variant filtering criteria were applied to enhance the accuracy and reliability of the data, the further validation of the functional effects of key variant sites in relevant functional genes is needed. Additionally, a deeper understanding of the genetic basis underlying the differences in equine traits is required. This study provides valuable foundational data and research ideas for exploring the genetic diversity of horse breeds, the domestication process, and the molecular mechanisms associated with economic traits. It also offers potential genetic markers for optimizing breeding strategies in horses.

## 5. Conclusions

Variant detection was performed on the whole-genome sequencing data of Mongolian Horses, Thoroughbreds, Xilingol Horses, and Grassland-Thoroughbreds. Principal component analysis, phylogenetic tree, and population structure analysis based on high-quality SNP data revealed that the Prairie Thoroughbred, Mongolian Horse, Xilingol Horse, and Thoroughbred can be classified into four distinct genetic groups, with the Grassland-Thoroughbred being more closely related to the Thoroughbred. LD decay analysis indicated that the Mongolian Horse exhibited the fastest decay rate, followed by the Grassland-Thoroughbred and Xilingol Horse, with the Thoroughbred showing the slowest decay rate. A total of 179 candidate genes were identified through SNP-based selective sweep analysis. These genes were mainly associated with traits related to exercise performance (*ATF2*, *NDUFS7*, *PRKG1*, *IGFN1*, *MTOR*, *TTN*) and growth and development (*MTOR*, *IGFN1*, COL24A1, *NEDD4*, *PIEZO1*). These genes may play an important role in the exercise performance and growth and development of the Grassland-Thoroughbred. This study reveals the genetic advantages and potential of the Grassland-Thoroughbred, aiming to enhance the overall performance of the breed. It fills the gap in the research on Grassland-Thoroughbreds and provides a scientific basis for future breeding improvements and breed enhancement.

## Data Availability

Sequence data that support the findings of this study have been deposited in the National Center for Biotechnology Information with the primary accession code PRJNA1253856.

## Supplementary Materials

The following supporting information can be downloaded at: https://www.mdpi.com/article/10.3390/ani15101462/s1, Figure S1: Breeding technology roadmap for the Grassland-Thoroughbred; Figure S2: The distribution of Indels on different chromosomes; Table S1: Information on the downloaded data in the present study; Supplementary; Table S2: DNA quality; Supplementary; Table S3: Quality control of sequencing results for different horse breeds; Table S4: ANNOVAR annotation results; Table S5: Gene annotation of top 1% outlier genomic regions from Fst and ratio; Supplementary; Table S6: Functional enrichment analysis of candidate genes.
