# Supplementary figures and images for: Preliminary Study on the Genetic Structure and Functional Candidate Genes of Grassland-Thoroughbreds Based on Whole-Genome Resequencing
Source: Animals (Basel). 2025 May 19;15(10):1462. doi: 10.3390/ani15101462 (PMC12108167; doi:10.3390/ani15101462)

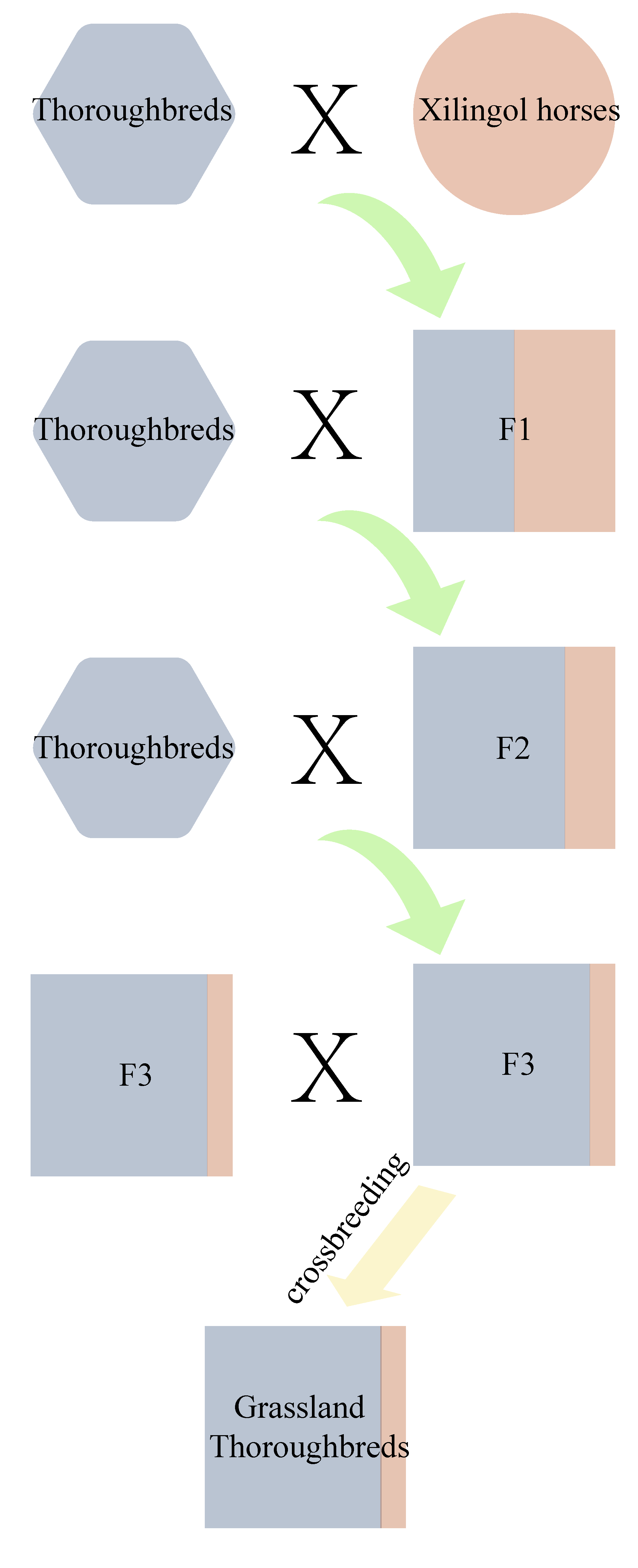

Supplement: Supplementary file 1 [file animals-15-01462-s001.zip › Supplementary Figure S1 Breeding technology roadmap for the Grassland-Thoroughbred.tif]
